# Vital sign monitoring with continuous pulse oximetry and wireless clinical notification after surgery (the VIGILANCE pilot study)—a randomized controlled pilot trial

**DOI:** 10.1186/s40814-019-0415-8

**Published:** 2019-02-26

**Authors:** James E. Paul, Matthew A. Chong, Norman Buckley, Prathiba Harsha, Harsha Shanthanna, Antonella Tidy, Diane Buckley, Anne Clarke, Christopher Young, Timothy Wong, Thuvaraha Vanniyasingam, Lehana Thabane

**Affiliations:** 1 Department of Anesthesia, McMaster University, Hamilton, Ontario Canada; 2 Western University, London, Ontario Canada; 3 Department of Anesthesia, Cumming School of Medicine, University of Calgary, Calgary, Alberta Canada; 4 St. Joseph’s Healthcare Hamilton, Hamilton, Ontario Canada; 5 Health Research Methods, Evidence and Impact, McMaster University, Hamilton, Ontario Canada

**Keywords:** Respiratory depression, Wireless respiratory monitoring, Pilot trial or study

## Abstract

**Background:**

Respiratory depression is a serious perioperative complication associated with morbidity and mortality. Recently, technology has become available to wirelessly monitor patients on regular surgical wards with continuous pulse oximetry and wireless clinician notification with alarms. When a patient’s SpO_2_ falls below a set threshold, the clinician is notified via a pager and may intervene earlier to prevent further clinical deterioration. To date, the technology has not been evaluated with a randomized controlled trial (RCT).

**Methods:**

We designed a parallel-group unblinded pilot RCT of a wireless monitoring system on two surgical wards in an academic teaching hospital. Postsurgical patients with an anticipated length of stay of at least 1 day were included and randomized to standard care or standard care plus wireless respiratory monitoring for up to a 72-h period. The primary outcomes were feasibility outcomes: average patients recruited per week and tolerability of the system by patients. Secondary outcomes included (1) respiratory events (naloxone administration for respiratory depression, ICU transfers, and cardiac arrest team activation) and (2) system alarm types and details. The analysis of the outcomes was based on descriptive statistics and estimates reported using point (95% confidence intervals). Criteria for success of feasibility were recruitment of an average of 15 patients/week and 90% of the patients tolerating the system.

**Results:**

The pilot trial enrolled 250 of the 335 patients screened for eligibility, with 126 and 124 patients entering the standard monitoring and wireless groups, respectively. Baseline demographics were similar between groups, except for slightly more women in the wireless group. Average patient recruitment per week was 14 95% CI [12, 16] patients. The wireless monitoring was quite tolerable with 86.6% (95% CI 78.2–92.7%) of patients completing the full course, and there were no other adverse events directly attributable to the monitoring. With regard to secondary outcomes, the respiratory event rate was low with only 1 event in the wireless group and none in the control group. The average number of alarms per week was 4.0 (95% CI, 1.6–6.4).

**Conclusions:**

This pilot study demonstrated adequate patient recruitment and high tolerability of the wireless monitoring system. A full RCT that is powered to detect patient important outcomes such as respiratory depression is now underway.

**Trial registration:**

ClinicalTrials.gov, Registration number NCT02907255, registered 7 September 2016—retrospectively registered.

## Background

Respiratory depression is a significant perioperative problem, associated with morbidity and mortality [1]. Several perioperative factors raise the risk for respiratory depression, including nurse-administered opioids, patient-controlled analgesia (PCA), and epidural analgesia [2]. Such opioid-induced respiratory depression is usually preceded by sedation and, if left untreated, can progress into cardiac arrest and death [3].

In settings without continuous monitoring, the incidence of respiratory depression varies depending on the definition used and the analgesic modality, but it has been reported to occur in about 1% of cases (Table 1) [2–8]. If one continuously monitored patients with oximetry and capnography, the incidence of respiratory depression can be as high as 12% [9]. The incidence of cardiac arrest and death from opioid overdose has been estimated at about 2 cases per 10,000 [10]. Given that the number of patients undergoing surgery annually is about 100 million worldwide, respiratory depression occurs between 1 and 12 million times and it results in about 20, 000 preventable deaths each year [11]. The troublesome aspect of this complication is that it can occur in healthy patients where the family and clinical staff do not anticipate a bad outcome [12].

**Table 1:** Critical incidents on acute pain services

Author Year	Schug [5] 1993		Sidebotham [6] 1997		Walder [7] 2001		Shapiro [2] 2005		Popping [3] 2008		Paul [8] 2013	
Methodology *N* (study)	Prospective cohort 3016		Prospective cohort 6035		Systematic review 1139		Prospective cohort 4500		Prospective cohort 18,925		Prospective cohort 27,550	
Analgesia modality *N* (cohort)	PCA 1111	PCA 5759	PCA 5759	Epidural	PCA 4114	Epidural	PCA 700	Epidural 680	PCA 1591	Epidural 14,223	PCA 18,406	Epidural 9144
Respiratory depression	3 (0.3%)	0 (0%)	14 (0.2%)	–	19 (4.6%)	–	13 (1.9%)	4 (0.6%)	63 (1.1%)	100 (0.7%)	135 (1.1%)	48 (0.7%)
Definition	Naloxone required		RR < 8 or SpO_2_% < 90		RR < 10 or SpO_2_% < 90		RR < 10		RR < 8 or SpO_2_% < 90		RR < 8 or SpO_2_% < 90	
Unresponsive/severe sedation	–	–	–	–	–	–	–	–	–	–	6 (0.03%)	3 (0.03%)
Death	0 (0%)	0 (0%)	0 (0%)	–	–	–	–	–	–	–	3 (0.02%)	1 (0.01%)

“–” not reported

The problem of unexpected respiratory depression among patients treated with opioids is compounded by the challenge of dealing with obstructive sleep apnea (OSA) patients in the perioperative period. OSA, characterized by the complete or partial obstruction of the upper airway during sleep, is common with about 25% of the general population (and a greater proportion of the surgical population) being at risk for this condition [13]. Patients with OSA have a higher risk of perioperative respiratory events, and consequently, the guidelines from the American Society of Anesthesiology recommend continuous monitoring of these patients [14, 15]. However, these guidelines are expert-based because no clinical trials have established the efficacy of continuous monitoring in this population.

These two problems (respiratory depression from opioids and obstructive sleep apnea) have caused significant logistic problems in hospitals, as many institutions do not have the equipment to provide continuous monitoring of patients outside of the intensive care units and there is limited capacity in the critical care areas to monitor all the patients at risk.

The impact of respiratory depression can be mitigated if it is recognized early enough and appropriate actions are taken to resuscitate the patient. Early recognition of this complication depends on frequent and regular vital sign assessments. However, such frequent monitoring may not be feasible on regular surgical wards because of a high patient to nurse ratio, particularly during the night shift. In this period, a patient can deteriorate from sedation to respiratory depression and ultimately cardiac arrest. Previously, cardiac monitoring with telemetry has been available and could be used for patients in the perioperative period. Although telemetry has been proven useful for certain groups of patients at risk for arrhythmias, this technology will only pick up cases of respiratory depression that progress into a cardiac event [16].

Recently, technology has become commercially available to continuously monitor pulse oximetry and notify clinical staff wirelessly via a paging system. The advantage of such a system is that clinicians can be notified immediately when a patient begins to decompensate from respiratory depression, with enough time to initiate resuscitation before the patient progresses to respiratory or cardiac arrest. Another benefit of these systems is that patients at risk can be monitored on regular surgical wards without utilizing additional nursing staff. A MEDLINE search (using the MESH terms: monitoring, oximetry, postoperative period, and clinical trial) was conducted to see if there was evidence to support the use of these systems. Only a single study was identified in the search that specifically investigated the postoperative period; a before-after study on a 36-bed orthopedic ward found that a respiratory monitoring system was effective in reducing the need for rescue resuscitations and ICU transfers [17]. Although there is a Cochrane review on pulse oximetry for perioperative monitoring, that systematic review only revealed studies that employed the monitoring in the postanesthetic care unit (PACU) or intensive care unit (ICU)—and not any studies exploring the utility of wireless respiratory monitoring on a regular surgical ward [1].

Given this paucity of robust trials, we conducted a single-center randomized pilot study to investigate the implementation of a wireless respiratory monitoring system versus standard care to prevent respiratory events in surgical patients for the first 72 h postoperatively. The primary aim of this pilot trial was to assess average patient recruitment per week and patient tolerance of the monitoring system (primary feasibility outcomes) and the number of alarms per week, the type of alarms, and the response to the alarm by the nursing staff (secondary feasibility outcomes).

Secondary clinical aims include to assess the effect of the intervention on the following clinical or process outcomes: respiratory events, which were defined as a composite of naloxone administration for respiratory depression; cardiac arrest team activation; and ICU transfers.

## Methods

### Setting and participants

Following research ethics approval by the Hamilton Health Sciences Research Ethics Board, this study was conducted on two surgical wards at the Juravinski Hospital in Hamilton, Ontario, Canada. These mixed surgical wards both have 24 beds, a nurse to patient ratio of 1:4, and about 1100 elective and emergent admissions for surgery per year. All adult surgical patients with planned or emergent admission to these two wards during the study period were considered to be eligible for inclusion into the study, if they had an anticipated length of stay of at least 24 h. The need for formal consent was waived by our Research Ethics Board, given that the wireless monitoring system was available as a standard monitor on the ward and of minimal risk to patients. Of course, patients that declined to participate were not enrolled in the study.

### Study design

This pilot study was randomized, parallel-grouped, unblinded, and controlled. Eligible surgical patients were randomized in a 1:1 ratio either to ward standard monitoring (control group) or to standard monitoring plus wireless respiratory monitoring (treatment group). The randomization sequence was computer-generated, and allocation was managed by the Research Coordinator of the Department of Anesthesia Research Office by marking up the master operating room (OR) list with the designation “Standard” or “Wireless” for all study patients that were being admitted to the applicable wards. This list was faxed to the charge nurses on the two surgical wards. Block randomization was used with no stratification. A variable block size of 2, 4, and 8 was used. It was not feasible to blind the patient or the caregivers to the treatment allocation, as the standard of care on the ward does not involve continuous pulse oximetry monitoring with a finger probe. Initial enrollment was performed by the clinical nurses on the ward, and a research nurse made daily rounds on the two surgical wards to ensure that study patients received the monitoring that they were allocated to by the randomization. In addition, they assisted the ward nurses if there were any technical issues with setting up the wireless monitoring system. The study was retrospectively registered with ClinicalTrials.gov (NCT02907255).

### Intervention

All patients received standard monitoring, which included vital sign assessments by the ward nurses every 4 h. A wireless respiratory monitoring system (Covidien Alarm Management System, Dublin, Ireland) was installed on two surgical wards. This system allows for bedside monitoring and wireless pager notification of clinical staff when an alarm threshold is exceeded. To reach a balance between patient safety and minimize false alarms, the alarm thresholds were set at SpO_2_ ≤ 90% and heart rate ≤ 50 or ≥ 130 per minute. To reduce the incidence of transient artifact-generated events, the bedside audio alarm and pager notification were both delayed for 15 s, which results in a 30-s interval before the nurse would be notified via pager. The attending physicians were also able to adjust the alarm thresholds in cases where patients have baseline abnormalities in their vital signs (that would exceed alarm thresholds) due to chronic disease.

Prior to implementing the wireless respiratory monitoring systems, nursing staff had in-service training sessions with the vendor and nurse educators to facilitate the incorporation of this wireless system into their clinical practice. The anesthetic technique used was left to the discretion of the case anesthesiologist.

### Outcomes

The primary feasibility outcomes were average patient recruitment rate per week and the percentage of patients that accepted and tolerated the monitoring system. The latter was defined as the ability of the patient to complete the full course of wireless monitoring, without asking to be withdrawn from the study. The criterion for success for this pilot study was an average recruitment rate of 14 patients per week and a patient compliance to the intervention of 85%. Secondary feasibility outcomes included the number of alarms per patient per day, the type of alarms, the response to the alarm by the nursing staff, and the reliability of the monitoring system. Secondary clinical and process outcomes included the respiratory event rate, which was defined as a composite of naloxone administration for respiratory depression; transfers to ICU; and cardiac arrest team activation. Patients were followed until discharge.

### Sample size and analysis

For the pilot phase of this study, a convenience sample of the first 250 patients enrolled was selected. Given that this was a pilot study, no formal sample size calculation was done but the authors felt that 250 patients would be sufficient to estimate recruitment and compliance rates as that would be sufficient to cover almost 20 weeks of recruitment. To calculate the sample size estimate for a full clinical trial, we used the reported event rate of 2% and assumed a 50% reduction with respiratory monitoring [2]. With *α* = 0.05 and power = 0.80, this corresponds to a total sample size of 2000 patients. If we achieve the target recruitment rate of 14 patients per week, we would be able to complete the full study sample size in about 36 months.

This study will be reported according to the CONSORT extension to pilot trials [18]. Data were analyzed by intention-to-treat basis. The baseline characteristics are analyzed using descriptive statistics reported as mean (standard deviation (SD)) for continuous variables and count (percentage) for categorical variables. We used descriptive statistics to report the results of feasibility outcome analyses. The criterion for statistical significance was set a priori at α = 0.05. All analyses of clinical and process outcomes are exploratory in nature. Analyses were performed using STATA 13.1 (StataCorp, College Station, Texas).

### Data management

Patient demographics, past medical history, surgery and anesthesia details, medications, postoperative complications, and patient specific alarm data were extracted from the patient’s chart and entered onto REDCap (Research Electronic Data Capture), which is a web-based data collection tool which is located on a secure server with a firewall maintained by Computer Services Unit (CSU) at McMaster. All data on REDCap was de-identified and collected by a trained study coordinator. Independently, a second study coordinator verified the accuracy of data collection. The alarm types and duration were downloaded from the Covidien monitoring systems onto an external encrypted hard drive, which was securely stored. Based on data from the alarm system vendors, we anticipated four alarms per patient per day, with half of them being false alarms. Hence, we predicted that nurses would have to document the details of an alarm approximately twice per day for each of their patients.

The respiratory event rate was collected via existing administrative systems in the hospital: the pharmacy department records naloxone administration for respiratory depression and the Department of Critical Care records all code blues and ICU transfers. Patient discharge summaries and physician’s progress notes were also reviewed to ensure the capture of all events. Missing data was handled by a secondary chart review by a separate reviewer to find the missing data whenever possible. In cases where the data truly could not be located, that case was omitted from the analysis for that variable.

### Equipment details and safety

This pilot study was possible for a relatively modest cost largely because the vendor supplied the hardware equipment for the respiratory monitoring systems for free and the study budget only had to cover the installation of the systems, the disposable oximetry probes, and the research-related expenses.

Our study included all patients admitted to either of the two surgical wards during the study period with an anticipated length of stay of at least 24 h. We anticipated that the majority of patients would accept the extra monitoring, as it was non-invasive and not associated with additional risk. Given the high patient volumes on both of the surgical wards, we did not expect to have significant problems with study recruitment.

The alarm threshold of 90% for the oximetry monitor was selected based upon clinical grounds and on feedback from the vendors. The 30-s delay in pager notification was chosen to reduce the number of false alarms. Research has shown that people adjust their behavior according to the perceived false alarm rate, and if the perception is that the alarms are unreliable, then they will only respond infrequently [19]. For comparison, the oximetry alarm threshold was set at < 80% in a Dartmouth study, and even with that threshold, there were 4.1 alarm alerts per patient per day with half of them considered nuisance alarms (e.g., “probe off of patient”) [17].

### Ethical considerations

We maintained confidentiality of all patient data and kept all case report forms in locked cabinets in the Department of Anesthesia Research office. Electronic data that was extracted from the monitoring systems was anonymized, as was all data in the final reports. Patients refusing to consent to the study were exempted.

## Results

**Fig. 1:**
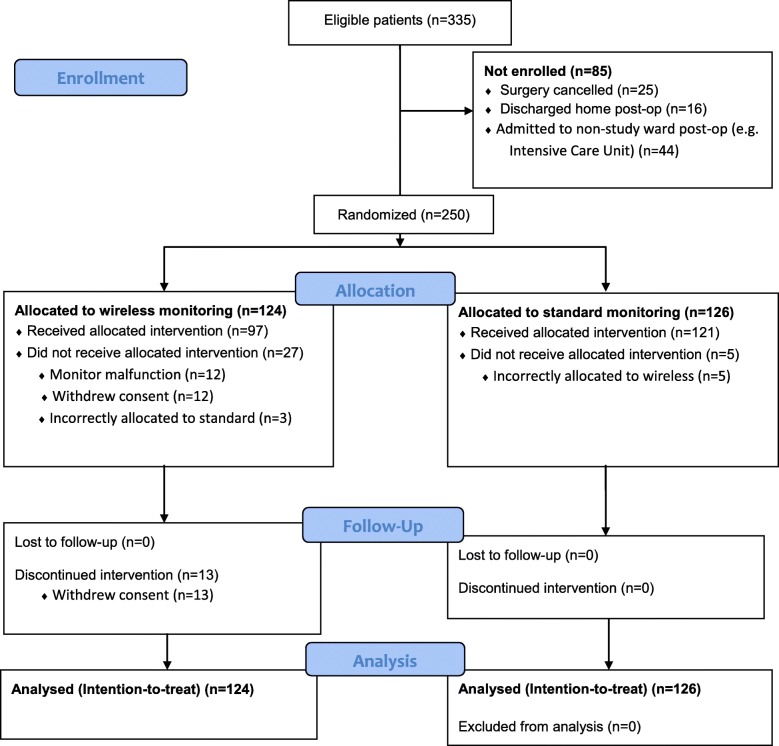
CONSORT Flowsheet

Of the 335 patients screened for eligibility, 250 were enrolled and randomized into the study (CONSORT Flowsheet, Fig. 1). Baseline demographics and ASA class distribution were similar between the groups, with the exception of slightly more female patients in the wireless monitoring group that received gynecologic surgery (Table 2).

**Table 2:** Baseline demographics. Numbers may not add up to 100% due to rounding

	Standard group	Wireless group
Total (*n*)	126	124
Age (years; mean (SD))	57.5 (15.8)	58.0 (13.9)
Sex (female; *n* (%))	78 (61.9%)	94 (75.8%)
ASA class (*n* (%))		
1	3 (2.4%)	0 (0%)
2	19 (15.1%)	29 (23.4%)
3	88 (69.8%)	75 (60.5%)
4	16 (12.7%)	20 (16.1%)
BMI (mean (SD))	27.5 (5.9)	28.6 (7.0)
OSA (*n* (%))	6 (4.8%)	4 (3.2%)
Type of surgery (*n* (%))		
General surgery	72 (57.1%)	46 (37.1%)
Urology	12 (9.5%)	12 (9.7%)
Gynecology	41 (32.5%)	65 (52.4%)
Orthopedics	0 (0%)	1 (0.81%)
Plastics	1 (0.79%)	0 (0%)
Type of anesthesia (*n* (%))		
General	89 (70.6%)	83 (66.9%)
General/Regional	30 (23.8%)	37 (29.8%)
Regional	7 (5.6%)	4 (3.2%)
Postoperative analgesia (*n* (%))		
PCA	61 (48.4%)	62 (50.0%)
Epidural	27 (21.4%)	37 (29.8%)
Multi-modal analgesia	33 (26.2%)	18 (14.5%)
Non-opioid multi-modal analgesia	5 (4.0%)	7 (5.6%)

### Primary feasibility outcomes

#### Average recruitment rate

The pilot study was conducted over 24 weeks from 7 October 2013 to 25 March 2014. As per institutional policy at Hamilton Health Sciences, this timeframe includes periods of decreased elective operating room activity during March break and the December holidays. Nonetheless, the average patient recruitment per week was 14 patients (95% CI 12–16). Naloxone administration for pruritus or respiratory distress was not considered as part of the composite primary outcome.

#### Tolerability of the wireless monitoring system

The wireless monitoring was quite tolerable among the 97 patients who received it, with 86.6% (95% CI 78.2–92.7%) of patients completing the full course of monitoring (either up to 72 h or until discharged from hospital). Of these 13 dropouts after initialization of the wireless monitoring, 10 patients did not provide an explicit reason for discontinuing their monitoring. The remaining three patients had baseline tachycardia (causing multiple alarms), multiple false alarms due to malfunctioning study hardware, and an in-hospital CPAP study, respectively.

There were no reported adverse events related to the monitoring system. Specifically, the finger probes did not cause any injuries and the mobile stand that housed the oximeter at the patient’s bedside was not involved in any patient falls or injuries.

### Secondary clinical and process outcomes

A respiratory event (naloxone administration for respiratory depression, transfers to ICU, or cardiac arrest team activation) occurred in 0 (out of 126 patients) in the standard group and 1 (out of 124 patients) in the wireless group (Table 3).

**Table 3:** Adverse respiratory events

	Standard monitoring	Wireless monitoring
Composite respiratory event rate	0	1
Naloxone use		
Pruritus	2	5
Respiratory depression	0	0
Respiratory distress	0	1
ICU transfer	0	1
Cardiac arrest team activation (code blue)	0	0

Wireless patients experienced 4.0 (95% CI 1.6–6.4) alarms per week. In total, 76.4% of the alarms were due to decreased SpO_2_%. The most common interventions applied by the nursing staff were applying oxygen, increasing the F_i_O_2_, rousing the patient, or encouraging deep breathing and coughing (Table 4). The remaining 23.6% of alarms were due to tachycardia secondary to pain or distress from nausea/vomiting.

**Table 4:** Alarm characteristics for patients on wireless monitoring

Alarm Characteristics	
Decreased SpO_2_	*n* (%)
Applied oxygen or increased F_i_O_2_	62 (50.4)
Roused patient or encouraged deep breathing and coughing	29 (23.6)
Called physician	3 (2.4)
Tachycardia	29 (23.6)
Total	123

## Discussion

Respiratory depression in the perioperative setting remains an important clinical problem with potentially preventable morbidity and mortality [1]. Herein, we report a pilot study for an RCT that is the first of its kind to investigate the use of an alarmed wireless respiratory monitoring system for the prevention of perioperative respiratory events on a regular surgical ward.

### Patient recruitment

We initially calculated that it would take a recruitment rate of about 15 patients per week to complete an adequately powered RCT in 3 years with 2000 subjects required. Although the weekly patient recruitment rate of 14 patients (95% CI 12–16) was near our target of 15, only 74.6% (250/335) of these subjects actually entered into the study (CONSORT Flowsheet). If we adjust for the proportion of patients actually enrolled and the recruitment rate from the pilot study, then it should take 4.4 years to complete a fully powered RCT on these two surgical wards.

### Patient tolerance of wireless monitoring

Only a small portion (12/124) of patients in the wireless group refused to consent to the wireless monitoring during allocation. Also, the wireless monitoring system was very tolerable among patients with 86.6% (84/97) subjects completing the course of monitoring.

### Reliability of the monitoring system

Of the 12 incidents of monitor malfunction that affected the wireless group, more than half (7/12) occurred within the first month of the pilot study. This high rate of crossover due to monitor malfunction motivated our study team to run additional training sessions for the wireless monitoring system to mitigate this issue. With time, the ward staff adapted well to the introduction of this new technology to the ward and the rate of these issues decreased substantially for the latter portion of the trial.

### Respiratory event rate

The respiratory event rate in this pilot study was lower than that reported in the literature [2, 7], with only 1/124 patients in the wireless group (and none in the standard group) experiencing an event. The study team is confident that no events were missed given that the full medical record of each patient was reviewed, as well as the existing administrative systems within the hospital that capture code blues, ICU transfers, and naloxone administration. The low event rate may be accounted for by several reasons. Some patients who were eligible for the study were sent directly to the intensive care unit (or observation unit) postoperatively, where deemed clinically necessary for patient safety. As this occurred prior to randomization, these patients never entered the trial. Nonetheless, this may have resulted in a healthier group of patients within the study. Another possibility is that this was a transiently low event rate due to the small sample size and the overall respiratory event rate might actually be what was predicted originally.

### Trial limitations

Blinding was deemed infeasible due to the visible nature of the intervention. Nonetheless, the primary and secondary outcomes for the pilot study were hard outcomes and unlikely to be influenced by knowledge of group allocation. Secondly, crossovers from the wireless group to the standard group were high (33.3% of patients; CONSORT Flowsheet). This pilot study enabled the study team to identify the etiology of these crossovers (e.g., 12/40 patients due to monitor malfunction) and take steps to mitigate them in a future full RCT. The other significant contributor to patient crossovers was withdrawing consent either prior to starting the wireless monitoring (12/40 patients) or after initialization (13/40 patients). We hope that better patient education strategies regarding the nature of the monitoring system and goals of the study will improve patient participation for the full course of monitoring. Lastly, although the hardware for this study was sponsored by the manufacturer, they played no role in the design, conduct, or synthesis of the trial.

This pilot trial was quite informative, and we learned a number of things that will help us conduct a full trial. While our recruitment rate was in range of our target, it will take over 4 years to reach our required sample size with that rate of recruitment. Originally, we only included patients with an expected length of stay of ≥ 3 days, and for the full trial, we will change that to ≥ 24 h and this should greatly increase our recruitment rate as a number of patients were excluded based on this criteria. To address patient acceptance of the respiratory monitoring system and adherence to the monitoring period, we plan to launch a nursing education initiative so that the ward nurses are better equipped to reassure patients regarding their concerns with the system. There were concerns with the reliability of the monitoring system, mostly related to its connection to the hospital’s wireless network. Going forward, we will have a research coordinator on sight every day (who liaises with the Biomedical Department and Hospital IT staff) to help address any of these issues as they arise.

## Conclusion

In summary, this pilot study of a wireless respiratory monitoring system showed that patient recruitment rate was adequate and the system had an excellent acceptance rate by patients and was well tolerated for the required monitoring period. As this pilot study was not powered to detect differences in the respiratory event rate, a full RCT of the wireless monitoring is underway. The full study of a wireless respiratory monitoring system will hopefully resolve the clinical equipoise surrounding the technology and patient outcomes. Positive results may facilitate more research in this technology and its use to reduce perioperative morbidity and mortality.

## Abbreviations

ICU: Intensive care unit
PCA: Patient-controlled analgesia
RCT: Randomized controlled trial
